# The Role of Leadership Level in College Students’ Decision-Making: Evidence From Event-Related Potential Analysis

**DOI:** 10.3389/fpsyg.2021.637323

**Published:** 2021-11-04

**Authors:** Yuwei Yang, Shunshun Du, Hui He, Chengming Wang, Xueke Shan, Huang Gu, Junfeng Zhao

**Affiliations:** ^1^School of Foreign Languages, Zhengzhou University of Aeronautics, Zhengzhou, China; ^2^Institute of Behavior and Psychology, Department of Psychology, Henan University, Kaifeng, China

**Keywords:** leadership, decision-making, Ultimatum Game (UG), Event-Related Potential (ERP), college students

## Abstract

Although risk decision-making plays an important role in leadership practice, the distinction in behavior between humans with differing levels of leadership, as well as the underlying neurocognitive mechanisms involved, remain unclear. In this study, the Ultimatum Game (UG) was utilized in concert with electroencephalograms (EEG) to investigate the temporal course of cognitive and emotional processes involved in economic decision-making between high and low leadership level college students. Behavioral results from this study found that the acceptance rates in an economic transaction, when the partner was a computer under unfair/sub unfair condition, were significantly higher than in transactions with real human partners for the low leadership group, while there was no significant difference in acceptance rates for the high leadership group. Results from Event-Related Potentials (ERP) analysis further indicated that there was a larger P3 amplitude in the low leadership group than in the high leadership group. We concluded that the difference between high and low leadership groups was at least partly due to their different emotional management abilities.

## Introduction

Leadership, which is commonly defined as a process of influence used in setting direction, building an inspiring vision, and creating something new to motivate organization members toward goal achievement (Yukl, 1999; Clarkson et al., 2019), is one of the most traditionally researched concepts in the behavioral sciences. Today, given that we live in highly complex social environments, a leader must not only be able to inspire followers to strive for organizational goals, but must also know how to handle conflicts in decision making. Making fast and accurate decisions has become an important hallmark of leadership (Westaby et al., 2010). Because of this, the leader’s risk decision-making ability has been a highly researched topic in the areas of management, economics, and academic psychology.

The topics of leadership and risk decision-making have been well studied in economics. Russ et al. (1996) found that low-performing sales managers behaved more avoidantly and irrationally in their decision-making while high performing managers made fast and accurate investment decisions to ensure a maximum investment return. Bahbouhi and Moussa (2019) observed that leadership positively affected the level of cooperation behavior in a public goods game. Furthermore, Loerakker and van Winden (2017) found emotional leadership played an important role in an intergroup conflict game experiment. All of these findings indicate the impact of leadership level on decision-making. However, there is a dearth of research to date exploring the difference in behavior and brain activity between high and low leadership level individuals when executing decision-making.

Previous studies have found that perception of fairness and emotional inhibition, like rationality, both have great impact on people’s decision making (De Cremer et al., 2003). A classic paradigm that involves both perception of fairness and emotional inhibition to examine social decision-making is the Ultimatum Game (UG) task (Güth et al., 1982, 2001). In this task, one of two players (the proposer) splits a sum of money into two parts; the other player (the responder) has the right to accept or reject the offer. If the offer is accepted, the money is allotted accordingly. If the responder rejects the offer, both players get nothing. Rational Choice Theory predicts that responders should accept any non-zero offers. However, empirical data have shown that people often reject unfair offers (i.e., the responder’s share of money is less than 20% of the total amount) (Camerer and Thaler, 1995; Hewig et al., 2011). Previous studies have used Event-Related Potentials (ERP) technology to explore the temporal course of UG tasks and have mainly focused on two representative components of feedback - the negative related component (FRN), and the P3 component. In these studies, FRN had a specific response to the valence of the results while P3 encoded both the valence and the magnitude (Philiastides et al., 2010). These two components were believed to be related to activities in the perception of fairness and the emotional inhibition system (Chen et al., 2019), so the impact of leadership level on UG performance should also be reflected in the ERPs.

In the current study, we aimed to explore high and low leadership level college students’ performance in the UG; concurrently, an ERP technique was utilized to examine brain activity during the decision-making process. We hypothesized that high and low leadership students would show some difference in both the behavioral index and ERPs during the UG task.

## Materials and Methods

### Participants

A total of 540 leadership questionnaires were distributed to undergraduates enrolled at Henan University and 488 valid questionnaires were obtained. Subjects were divided into high and low leadership groups (the upper and lower 7% of the score distribution) according to the total score on the leadership questionnaire. Finally, 67 subjects participated in the follow-up UG experiment (high leadership group: *n* = 35, female = 20, mean_age_ = 18.74, *SD*_age_ = 1.039; low leadership group: *n* = 32, female = 17, mean_age_ = 19.25, *SD*_age_ = 0.950). The number of males and females was closely balanced and age-matched. All of the participants were right-handed, had normal or correct-to-normal vision, and reported no history of psychiatric disorders. All participants provided written informed assent and consent before the experiment. Each participant received a nominal amount of money at the completion of the experiment as a token of appreciation. The study protocol was approved by the Institutional Review Board of Henan Provincial Key Laboratory of Psychology and Behavior.

### Tools

The study used the Student Leadership Practices Inventory (SLPI) to measure the level of college students’ leadership level. Students assessed how frequently they engaged in a range of behavioral practices using a five-point Likert scale ranking each of the 30 items in the inventory from 1 to 5 (where 1 indicates “rarely” and 5 indicates “frequently”); responses were further categorized into five dimensions of exemplary leadership: model the way, inspire a shared vision, challenge the process, enable others to act, encourage the heart. Total responses for each behavioral practice had a range from 6 to 30, which represented the sum of the response scores for each of the six behavioral statements related to that behavioral practice. Students’ responses on all 30 items were summed to create a composite scale for leadership. In terms of reliability, the Cronbach’s alpha coefficient of internal reliability in the current study was 0.94. The SLPI demonstrates a reasonably robust validity across multiple student populations (Posner and Brodsky, 1992).

### Stimuli and Procedures

Participants were instructed to play the UG which was computerized by E-prime 2.0 (Psychology Software Tools Inc., Pittsburgh, PA, United States). The experimental task consisted of four blocks for a total of 288 offers, each involving 100 Y– split. These offers fell into one of the three levels of fairness - fair offer (50 Y–, 50 Y–), sub unfair offer (60 Y–, 40 Y–; 70 Y–, 30 Y–), and unfair offer (80 Y–, 20 Y–; 90 Y–, 10 Y–). In the first two blocks, the participants were told that the proposals were put forward by anonymous college students, while during the last two blocks, the participants were told their partner was a computer. The stimuli at each block contained 24 (50 Y–, 50 Y–) allocation trials, 12 (90 Y–, 10 Y–) allocation trails, 12 (80 Y–, 20 Y–) allocation trails, 12 (70 Y–, 30 Y–) allocation trails, and 12 (60 Y–, 40 Y–) allocation trails.

To reflect the authenticity of the experiment, subjects (the responders) were told that they would be playing the UG experiment over a computer network with anonymous participants. In fact, no one joined the game. At the same time, the participants were told that the monetary reward they would receive after the experiment depended on their choice of the allocation during the experiment.

**FIGURE 1:**
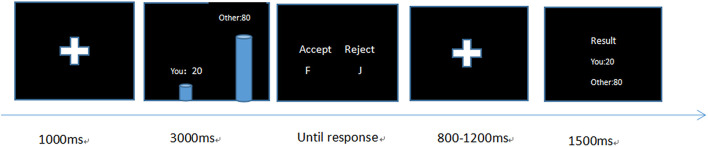
Procedures of the ultimatum game (UG) task.

Each trail in the task commenced with a fixed time that lasted for 1000 ms. After this fixed time, a picture of an offer was displayed on the screen for 3000 ms. During this period, the subjects were required to respond to the presented offer by pressing “F” for acceptance and “J” for rejection on a keyboard. After this, a fixed time lasting randomly from 800 to 1200 ms, feedback results were presented on a central screen for 1500 ms, after which the task proceeded to the next trail. If the participant accepted, the proposal was implemented; if the responder rejected the proposals, both parties received nothing (see Figure 1).

### Data Management and Analysis

Participant electroencephalogram (EEG) signals were recorded from a 32 scalp standard channel cap (Brain Products, Munich, Germany) with electrodes positioned as per the international 10/20 system. The vertical electrooculogram (VEOG) was recorded with the electrode located above and below the right eye, the horizontal electrooculogram (HEOG) was recorded with the left and right electrodes located at 1.5 cm opposite the left eye. All electrode recordings were referenced online to FCz. All inter-electrode impedances were controlled below 10 KΩ. The signals were amplified using a 0.01–100 Hz band-pass filter and the sampling frequency was at 500 Hz/channel.

BrainVision Analyzer 2.0 software (Brain Products, Munich, Germany) was used for off-line analysis and the Brain Product Extension Toolbox was used for processing EEG data. For each subject, EEG waves were re-referenced to the average of the bilateral mastoid signals and filtered with a band-pass filter (0.01–30 Hz) to lessen residual high-frequency artifacts in the signals. Epochs were extracted 200 ms before and 800 ms after the presentation of stimuli. The pre-stimulus time interval (−200 ms to stimuli onset) was utilized as a baseline correction for each epoch. ERPs were averaged separately according to the experimental design. After excluding the EEG data with obvious myoelectricity and drift, SPSS Statistics 20.0 (IBM, Somers, United States) was used for statistical analysis of the data.

Based on previous studies and visual inspection of the topographies in the current study, relevant electrodes and time windows were selected for analysis. Specifically, FRN (260–310 ms) at F3, F4, and FZ; P3 (350–550 ms) at P3, P4, and PZ.

### Experimental Design

The acceptance rate was analyzed using a three-way offer (unfair, sub unfair vs. fair) × partner (computer vs. human) × leadership (high leadership vs. low leadership) ANOVA test, while the above-mentioned electrodes were added into the analyses on the FRN and P3 amplitudes as the fourth factor. The Greenhouse-Geisser correction for ANOVA tests was used when appropriate. *Post hoc* comparisons were conducted using the Bonferroni correction.

## Results

### Behavioral Results

**FIGURE 2:**
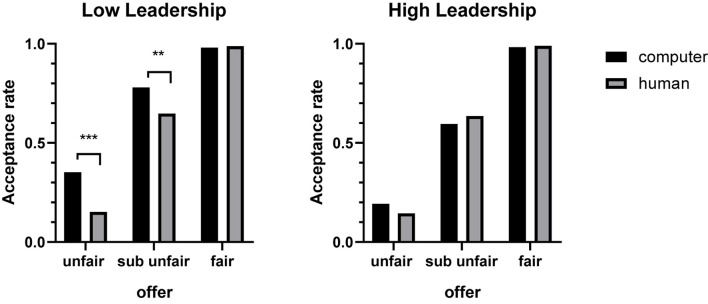
The acceptance rate for three types of the offer between Low leadership and High leadership groups under human/computer conditions. **p < 0.01, ***p < 0.001.

Behavioral results indicated a significant difference between type of partner acceptance rate [*F*_(__1_,_65__)_ = 5.508, *p* = 0.022, η*^2^* = 0.078], with the acceptance rate of the offer in computer condition (64.7 ± 2.4%) being higher than that of the human partner condition (59.3 ± 2.0%). The main effect of the offer was significant [*F*_(__2_,_130__)_ = 259.395, *p* < 0.001, η*^2^* = 0.800]: the acceptance rate of a fair offer (98.5 ± 5.0%) was higher than that of an unfair offer (21.0 ± 3.6%) and sub unfair offer (66.4 ± 3.0%). There was a significant interaction between the leadership level and partner [*F*_(__1_,_65__)_ = 5.393, *p* = 0.023, η*^2^* = 0.077]. There was no significant difference in acceptance rate whether the partner was a human (59.0 ± 3.3%) or computer (59.0 ± 2.8%) in the high leadership group. However, there was a significant difference in the low leadership group, with the acceptance rate in the computer condition (70.4 ± 3.5%) being higher than that in the human condition (59.6 ± 2.9%). Finally, partner × offer × leadership triple interaction was significant [*F*_(__2_,_130__)_ = 6.546, *p* = 0.039, η*^2^* = 0.049]. The low leadership group showed higher acceptance rates when the partner was a computer (35.3 ± 7.5%/77.9 ± 5.3%) relative to being a human (15.2 ± 5.7%/64.8 ± 6.0%) in unfair/sub unfair offers. There was no significant difference in this regard between computer and human condition in the high leadership group (see Figure 2).

### Event-Related Potentials Results

#### Feedback-Related Negative

**FIGURE 3:**
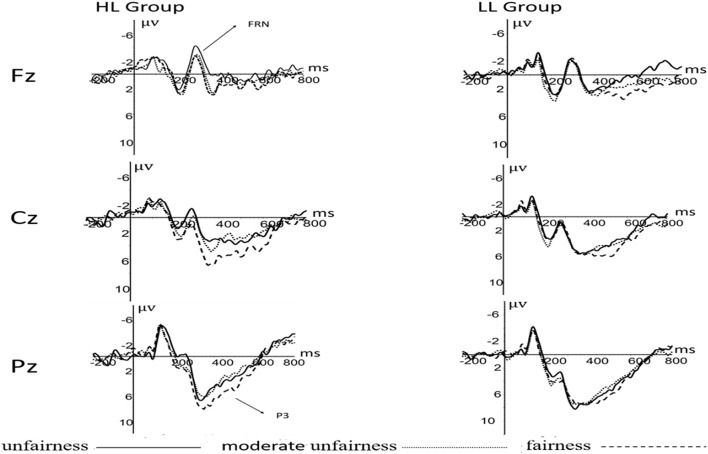
P3, FRN components at Fz, Cz, Pz electrodes when the partner is human.

Results showed a significant difference in electrode readings [*F*_(__2_,_126__)_ = 28.186, *p* < 0.001, η*^2^* = 0.309]. The amplitude of FRN at F3 was smaller than that at F4 and FZ (*P* < 0.01). There was an interaction between the electrode readings and the offer [*F*_(__4_,_252__)_ = 18.928, *p* < 0.001, η*^2^* = 0.231]. The amplitude of FRN at F3 and FZ was significantly different in an unfair offer, with the amplitude at F3 is greater than that at FZ (*P* < 0.001). However, there was no such difference in sub unfair and fair offer (*P* > 0.05) (see Figure 3).

#### P3

**FIGURE 4:**
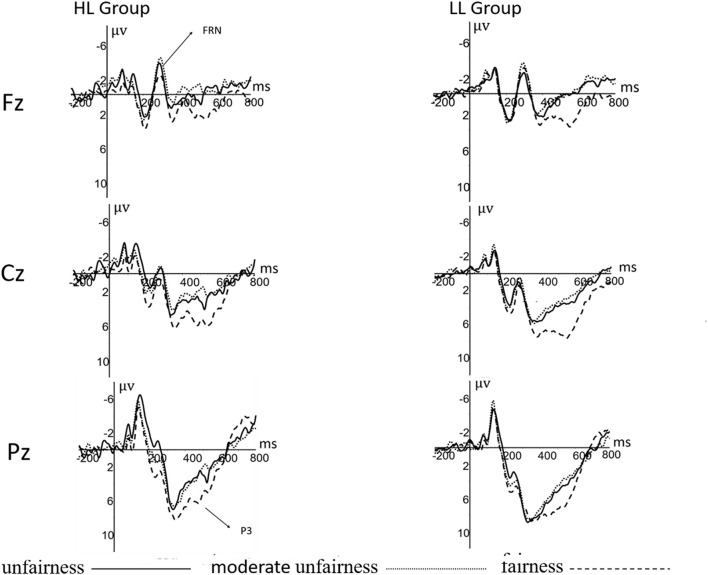
P3, FRN components at Fz, Cz, Pz electrodes when the partner is computer.

There was a significant difference between leadership groups [*F*_(__1_,_63__)_ = 4.618, *P* = 0.035, η*^2^* = 0.068]. The amplitude of P3 in low leadership group (5.6 ± 0.6) was larger than that in high leadership group (3.8 ± 0.6). Results also showed a significant difference in electrode readings [*F*_(__2_,_126__)_ = 21.242, *P* < 0.001, η*^2^* = 0.252]. The amplitude at P4 point was larger than that at P3 and PZ (*P* < 0.01). The main effect of an offer was significant [*F*_(__2_,_126__)_ = 10.012, *P* < 0.001, η*^2^* = 0.137]. The amplitude of P3 induced by a fair offer was larger than that of an unfair offer and a sub unfair offer (*P* < 0.01) (see Figure 4).

## Discussion

### Leadership and Acceptance in Ultimatum Game

This study employed the classic UG paradigm in conjunction with ERP to provide evidence for a neural underpinning of decision-making between college students with different leadership levels. In regards to the behavioral results, consistent with previous studies (Güth et al., 1982; Sanfey et al., 2003; van’t Wout et al., 2010), we found differences in acceptance rate among three levels of fairness: With a decrease in fairness, the acceptance rate of the offer decreased. The hypothesis of the “rational man,” where humans seek the maximum benefit under uncertain circumstances, can be used to explain these results. In addition, an unfair distribution proposal will cause a strong negative emotion in the responder, and, in turn, this emotion influences the acceptance rate of unfair offers. Pillutla and Murnighan (1996) hypothesize that, driven by this negative emotion, an individual chooses to reject the unfair offer. In the present study, the negative emotion of the responders should therefore become more and more intense with the increasing degree of inequity; in this study, the acceptance rates under unfair and sub unfair offers were in fact lower than those for a fair offer.

We also found evidence that different levels of leadership influenced the acceptance rate of offers in the UG. There was no significant difference in the acceptance rate whether the partner was human or computer when subjects were in the high leadership level category. However, the acceptance rate of low leadership subjects under computer condition was higher than when the partner was human. The different acceptance rates of partners can be attributed to emotional control - individuals in the high leadership group demonstrated a better ability in term of emotional inhibition, and were able to better manage negative emotions (Behrman and Perreault, 1984). Therefore, regardless of whether the partner was human or computer, students in the high level leadership group demonstrated reduced emotional reaction in the decision making process. By contrast, low leadership subjects appeared to lack strong emotional control ability. Therefore, when they understood that their partner was a computer, they tended to attribute the proposal more to random selection. However, when they understood that their partner was human, their decision-making behavior was potentially affected by many factors such as social factors, emotional fluctuations, etc (Sanfey et al., 2003; Rilling and Sanfey, 2011), and the unfair offer was more likely to be attributed to being man-made. In addition, the study found that the acceptance rate of the unfair/sub unfair offers in the computer condition was significantly higher than in the human condition in the low leadership group, while there was no significant difference in the high leadership group. That is to say, individuals with low leadership were inclined to risk preference under negative emotions in the risk decision-making task due to the lack of a strong emotional inhibition ability.

### Leadership and P3 in Ultimatum Game

This study also found significant differences in P3 between the two groups. The P3 is mainly activated in the parietal lobe of the brain (Christopoulos et al., 2009) and has been associated with the individual’s emotional involvement, which is related to attention bias, emotional arousal, and new and different stimuli. We hypothesized that the higher the level of emotional arousal, the larger the amplitude of P3. To this end, we recorded EEG from subjects playing the role of the responders in the UG. The results indicate that the amplitude of P3 in the low leadership level group was larger than that in the high leadership group. In addition, P3 amplitude is considered to represent the motivation and emotional significance of stimulation (Martin and Potts, 2004; Gu et al., 2011). The subjects with low leadership have weaker emotional inhibition ability and respond strongly to stimulation, thus producing a larger P3 amplitude than high leadership group. Another result of this study showed that the P3 amplitude induced under a fair offer was larger than that induced by unfair and sub unfair offer conditions, which is consistent with the previous research results (Hewig et al., 2011). P3 showed greater sensitivity in the positive condition than negative condition and may explain the relation between rejection rates and P3 following different fair level offers - the subjects were more likely to be affected by task-related positive feedback. Therefore, no matter whether the partner was human or computer, participants usually allocated more attention resources to positive stimulus, that is, to a fair offer.

### Leadership and Feedback-Related Negative in Ultimatum Game

The neural activity corresponding to the Feedback-Related Negative (FRN) wave is mainly distributed in the prefrontal lobe of the brain. Some studies of FRN suggest that the activation degree of FRN under unfair conditions is higher than that under fair conditions in UG tasks, which indicates that FRN is more sensitive to negative results than positive results (Boksem and De Cremer, 2010; Campanha et al., 2011; Van der Veen and Sahibdin, 2011; Alexopoulos et al., 2012). This is in contrast to studies that have found that FRN is not sensitive to negative results. This may be due to the fact that FRN mainly reflects the evaluation of the potency of results. From this perspective, the presentation style will affect the activation of FRN. In the design of the experimental process of this study, the order of stimulation subjects received was partner, offer, and result valence. According to Kaniman’s Dual-Process Theory, the participants’ attention resources may be mainly occupied by the partner in a limited reaction time situation (Kahneman and Tversky, 2013). In this case, subjects often make imperfect decisions to deal with the challenges presented (Rahman et al., 2001; Slovic et al., 2005), culminating in the results exceeding expectations. Another possible explanation is that FRN is greatly affected by individual differences. In this study, college students aged 18–23 were selected as subjects. The cohort was young, well-educated, and generally of high psychological stability. Therefore, even in the face of negative events, there might not be too much emotional fluctuation within this group. Consequently, when they were faced with different allocation conditions, there was no significant difference in the results of the strategy (Martin and Potts, 2004; Foti and Hajcak, 2009; Gu et al., 2010; Onoda et al., 2010).

## Conclusion

This experiment is the first to use the UG task to explore the differences of the temporal course of the brain in risk decision-making of high and low leadership level college students. The sense of fairness was one of the foremost factors affecting decision-making; the lower the fairness, the greater likelihood of rejection. Secondly, there were differences in decision-making among college students with different leadership levels, which may be related to their abilities of emotional inhibition and control. However, because emotion was not used as a variable in this study, more direct evidence is needed to verify this finding.

## Data Availability Statement

The raw data supporting the conclusions of this article will be made available by the authors, without undue reservation.

## Ethics Statement

The studies involving human participants were reviewed and approved by the Institutional Review Board of Henan Provincial Key Laboratory of Psychology and Behavior. The participants provided their written informed consent to participate in this study.

## Author Contributions

JZ, HG, SD, and YY: substantial contributions to the conception or design of the work and final approval of the version to be published. HG, SD, and HH: acquisition, analysis, or interpretation of data for the work. SD, CW, and XS: drafting the work or revising it critically for important intellectual content. JZ, HG, and SD: agreement to be accountable for all aspects of the work in ensuring that questions related to the accuracy or integrity of any part of the work are appropriately investigated and resolved. All authors contributed to the article and approved the submitted version.

## Conflict of Interest

The authors declare that the research was conducted in the absence of any commercial or financial relationships that could be construed as a potential conflict of interest.

## Publisher’s Note

All claims expressed in this article are solely those of the authors and do not necessarily represent those of their affiliated organizations, or those of the publisher, the editors and the reviewers. Any product that may be evaluated in this article, or claim that may be made by its manufacturer, is not guaranteed or endorsed by the publisher.
