# Supplementary material for: The Role of Leadership Level in College Students’ Decision-Making: Evidence From Event-Related Potential Analysis
Source: Front Psychol. 2021 Nov 4;12:637323. doi: 10.3389/fpsyg.2021.637323 (PMC8600661; doi:10.3389/fpsyg.2021.637323)

Questionnaire

大学生领导力实践行为调查量表

“学生领导力实践量表”（SLPI）是一个关于大学生领导力实践行为的评估量表。下面每一个问题都有五个选项，请您从中选取最符合您真实想法和情况的一项，在选中的数字上直接打√。

| 题 目 | 从不 | 很少 | 有时 | 经常 | 总是 |
| --- | --- | --- | --- | --- | --- |
| 1.对于自己期待他人做到的事，我会以身作则。 | 1 | 2 | 3 | 4 | 5 |
| 2.我会展望未来，并就那些我看到的、未来可能会对我们产生影响的事情，与他人进行沟通。 | 1 | 2 | 3 | 4 | 5 |
| 3.我会寻找各种途径来发展和挑战自己的潜能。 | 1 | 2 | 3 | 4 | 5 |
| 4.在工作过程中，我更愿意培育合作而非竞争关系。 | 1 | 2 | 3 | 4 | 5 |
| 5.我表扬出色完成工作任务的人。 | 1 | 2 | 3 | 4 | 5 |
| 6.我花时间和精力确保团队成员遵守大家已经达成共识的原则和规范。 | 1 | 2 | 3 | 4 | 5 |
| 7.我向团队成员阐述我们应当有能力达到的目标。 | 1 | 2 | 3 | 4 | 5 |
| 8.我努力寻求各种方法鼓励大家创新。 | 1 | 2 | 3 | 4 | 5 |
| 9.我积极听取各种不同的意见。 | 1 | 2 | 3 | 4 | 5 |
| 10.我鼓励团队中的成员。 | 1 | 2 | 3 | 4 | 5 |
| 11.我在组织中信守承诺。 | 1 | 2 | 3 | 4 | 5 |
| 12.我和组织成员分享对于组织正向发展的愿景。 | 1 | 2 | 3 | 4 | 5 |
| 13.对那些可能会对组织产生影响的事件和活动，我始终保持关注。 | 1 | 2 | 3 | 4 | 5 |
| 14.我尊重他人。 | 1 | 2 | 3 | 4 | 5 |
| 15.我不仅为组织中的成员提供支持，同时也对他们的贡献表示肯定和赞赏。 | 1 | 2 | 3 | 4 | 5 |
| 16.对我的行为如何影响他人的表现这一问题，我寻求各种途径来获得反馈。 | 1 | 2 | 3 | 4 | 5 |
| 17.我与他人讨论如何通过朝向共同的目标努力来实现双赢。 | 1 | 2 | 3 | 4 | 5 |
| 18.当事情的发展出乎我们意料时，我提问自己：“我们可以从这些经验中学到什么？”。 | 1 | 2 | 3 | 4 | 5 |
| 19.我支持组织中的其他成员独立进行决策。 | 1 | 2 | 3 | 4 | 5 |
| 20.对这些遵守我们价值观的人，我会特别留意对他们公开地表示认可。 | 1 | 2 | 3 | 4 | 5 |
| 21.我在团队共识基础上建立组织的价值观。 | 1 | 2 | 3 | 4 | 5 |
| 22.当讨论到那些我们组织孜孜以求的目标时，我总是乐观而积极。 | 1 | 2 | 3 | 4 | 5 |
| 23.对于要着手的项目，我确保我们已经设定了目标并制定了详细的计划。 | 1 | 2 | 3 | 4 | 5 |
| 24.我给他人很大的自由和选择，让他们自己决定如何开展工作。 | 1 | 2 | 3 | 4 | 5 |
| 25.我寻求各种方式让大家一起来庆祝成功。 | 1 | 2 | 3 | 4 | 5 |
| 26.我会与他人谈论那些指引我行动的价值观和原则。 | 1 | 2 | 3 | 4 | 5 |
| 27.我相信我们所做的是为了更高的目标，很有意义和价值，每当谈及此时，我都会非常坚定。 | 1 | 2 | 3 | 4 | 5 |
| 28.我勇于尝试在组织中以不同的方式开展工作。 | 1 | 2 | 3 | 4 | 5 |
| 29.我为他人提供承担领导责任的机会。 | 1 | 2 | 3 | 4 | 5 |
| 30.我确信在我们的组织中每个人都能够因他们的贡献得到创造性的认可。 | 1 | 2 | 3 | 4 | 5 |

请检查一下，正反面是否全部做完或者是否有漏答！

如果您愿意继续参加接下来的研究项目，请留下任何可以联系到您的联系方式：

1.手机号码： QQ号：

2.如果您需要什么帮助，请留写明诉求：

**请把问卷交给相关负责人 再次感谢您的认真作答！**

Institutional Review Board approval


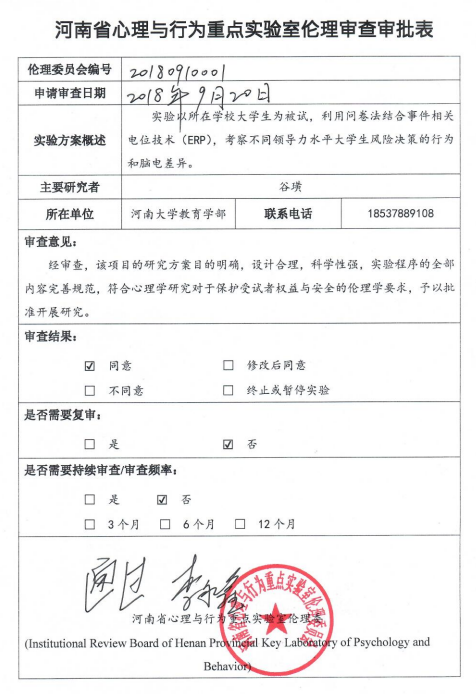

Supplement: Supplementary file 1 [file Table_1.DOCX]
